# Acute symptomatic seizures in the emergency room: predictors and characteristics

**DOI:** 10.1007/s00415-021-10871-5

**Published:** 2021-11-02

**Authors:** Lili C. S. Reinecke, Jakob I. Doerrfuss, Alexander B. Kowski, Martin Holtkamp

**Affiliations:** 1 Department of Neurology with Experimental Neurology, Charité-Universitätsmedizin Berlin, Corporate Member of Freie Universität Berlin, Humboldt-Universität zu Berlin and Berlin Institute of Health, Berlin, Germany; 2 Epilepsy-Center Berlin-Brandenburg, Institute for Diagnostics of Epilepsy, Berlin, Germany

**Keywords:** Alcohol withdrawal, Antiseizure medication, Epilepsy, Stroke, Unprovoked seizure

## Abstract

**Background:**

When treating patients with epileptic seizures in the emergency room (ER), it is of paramount importance to rapidly assess whether the seizure was acute symptomatic or unprovoked as the former points to a potentially life-threatening underlying condition. In this study, we seek to identify predictors and analyze characteristics of acute symptomatic seizures (ASS).

**Methods:**

Data from patients presenting with seizures to highly frequented ERs of two sites of a university hospital were analyzed retrospectively. Seizures were classified as acute symptomatic or unprovoked according to definitions of the International League Against Epilepsy. Univariate and multivariate analysis were conducted to identify predictors; furthermore, characteristics of ASS were assessed.

**Results:**

Finally, 695 patients were included, 24.5% presented with ASS. Variables independently associated with ASS comprised male sex (OR 3.173, 95% CI 1.972–5.104), no prior diagnosis of epilepsy (OR 11.235, 95% CI 7.195–17.537), and bilateral/generalized tonic–clonic seizure semiology (OR 2.982, 95% CI 1.172–7.588). Alcohol withdrawal was the most common cause of ASS (74.1%), with hemorrhagic stroke being the second most prevalent etiology. Neuroimaging was performed more often in patients with the final diagnosis of ASS than in those with unprovoked seizures (82.9% vs. 67.2%, *p* < 0.001). Patients with ASS were more likely to receive acute antiseizure medication in the ER (55.9% vs. 30.3%, *p* < 0.001).

**Conclusions:**

In one quarter of patients presenting to the ER after an epileptic fit, the seizure had an acute symptomatic genesis. The independently associated variables may help to early identify ASS and initiate management of the underlying condition.

## Introduction

Epileptic seizures are among the most common neurological conditions leading to presentation in the emergency room (ER) [1]. With respect to rough etiological groups, epileptic fits are dichotomized into acute symptomatic seizures (ASS) and unprovoked seizures (US). ASS are defined to occur in close temporal and likely causal relationship to an acute cerebral or systemic impairment of structural, metabolic, toxic, infectious or inflammatory origin [2]. The conceptual reason why ASS and US are differentiated is long-term prognosis regarding seizure recurrence [3]. A patient with an US and risk of at least 60% to develop seizures over the following decade is considered to have epilepsy. This 60% threshold is reached when a second US occurs > 24 h apart from the first US or if EEG and/or MRI findings after the first US indicate a significantly elevated risk of seizure recurrence [4]. In contrast, ASS have a lower long-term recurrence risk which is approximately 20% in the 10 years after the index seizure [3].

When treating a patient after a seizure in the ER, rapidly discerning ASS from US is pivotal as the former may indicate the necessity of imminent management of the potentially life-threatening underlying condition. Separating ASS from a first US is also important in a long-term perspective as administration of antiseizure medication (ASM) is handled differently and as the two conditions bear different psychosocial consequences, such as duration of driving ban [5].

In this study, we sought to evaluate predictors for ASS compared to US in patients presenting to the ER with an epileptic fit. As secondary outcome parameter, we aimed to compare the diagnostic findings and the acute pharmacological management of patients with ASS and US.

## Methods

### Patient sample

In this retrospective analysis, patients who presented to the ERs of two sites of the Charité-Universitätsmedizin Berlin (Campus Virchow-Klinikum and Campus Mitte) from 1st of January 2014 to 31st of December 2014 with the diagnosis of a seizure or epilepsy (ICD-10 codes G40.x and R56.x) were examined. Patients aged 18 years or older with a confirmed diagnosis of an epileptic seizure as the leading cause for presenting in the ER were included in this study. We did not include patients who presented with status epilepticus (G41.X). The rationale behind this decision was to keep the study population more homogenous as status epilepticus differs from self-limiting seizures with respect to the spectrum of causes as well as with respect to clinical management and prognosis [6]. In case of more than one seizure-related visit per patient to the ER in the study period, only the first visit was taken into account. This was done to avoid distortion of our results regarding demographics when including a patient with seizure recurrence more than one time. Data on demographics and seizure characteristics, diagnostic procedures and pharmacological treatment were retrieved from the in-house database. To validate the initially given diagnosis, documentation from the ER and discharge reports were analyzed by two neurologists with expertise in epileptology (LSR and ABK). The study was approved by the local ethics committee (EA1/061/15) and, therefore, has been performed in accordance with the ethical standards laid down in the 1964 Declaration of Helsinki and its later amendments. Due to the retrospective nature of the study, informed consent from individual patients was waived.

### Definitions and classifications of variables

ASS were defined according to the International League Against Epilepsy (ILAE) recommendations as a seizure occurring in close temporal relationship with an acute systemic or central nervous system insult [2]. ASS were categorized into those occurring within 48 h of metabolic–toxic disturbances and those manifesting within 7 days of acute CNS lesions due to structural or infectious/inflammatory pathology. ASS was considered a consequence of alcohol withdrawal if the seizure occurred within 48 h of last alcohol consumption in patients with alcohol addiction; in addition, typical neuropsychiatric and vegetative signs were considered if present. Concomitant etiologies such as dysionias were excluded via blood testing. US were defined as occurring in the absence of the mentioned acute metabolic–toxic disturbances or CNS insults. In accordance with the current ILAE recommendations, seizures due to facilitating factors in patients with established epilepsy such as non-adherence to ASM or sleep deprivation were not considered as acute symptomatic [2]. Based on semiology, seizures were dichotomized into bilateral/generalized tonic–clonic seizures (i.e., focal to bilateral tonic–clonic seizures as well as tonic–clonic seizures with generalized and unknown onset) and focal seizures with preserved or impaired awareness. Patients were classified to have had an unclassified seizure semiology, if seizure description did not allow attribution to a specific semiology.

### Statistical analysis

Categorical data were analyzed with Pearson’s Chi-Square Test. For continuous data, the median and the interquartile range (IQR) are reported. For group comparison, the Mann–Whitney *U* test was used for continuous nonparametric variables. Logistic regression analysis was performed to calculate odd ratios with 95% confidence intervals (95% CI) for identification of variables being independently associated with ASS (inclusion method: stepwise backward, *p* < 0.1 [p in], *p* < 0.05 [p out], iteration 20, cutoff set 0.26, constant incorporated). In the logistic regression model, relevant clinical data obtainable upon examination in the ER (i.e., patients’ sex and age, seizure semiology and absence vs. presence of prior diagnosis of epilepsy) were included as possible confounding variables. *p* values < 0.05 were considered statistically significant. Statistical analysis was performed with SPSS Statistics, version 26, (IBM, Armonk, US-NY).

## Results

### Study population

**Fig. 1:**
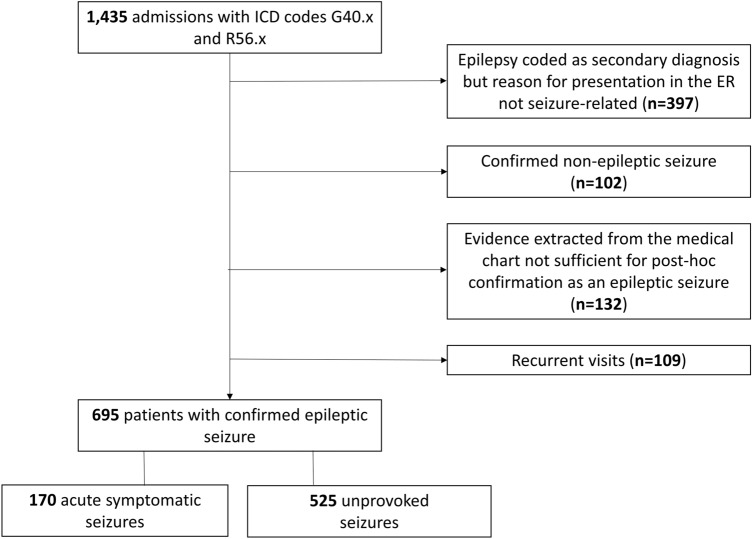
Patient selection. This figure shows the patient selection according to inclusion and exclusion criteria as well as allocation of patients with regards to acute symptomatic and unprovoked seizures

During the study period, 1435 adult patients admitted to the ER had been coded with epileptic seizures or epilepsy (ICD-10 codes R 56.8 and G40.x). Of those, 824 admissions were excluded from the analysis. The patient selection is shown in Fig. 1.

Our final analysis included 695 patients, 452 (35%) were female, median age was 47.8 years (IQR 32.0–62.9), 213 patients (30.6%) presented with a first seizure. One hundred seventy patients (24.5%) had an ASS and 525 patients (75.5%) had an US.

### Predictors for ASS

Binary logistic regression showed male sex (OR 3.173), no prior diagnosis of epilepsy (OR 11.235), and bilateral/generalized tonic–clonic seizures (OR 2.982) to be independently associated with ASS (see Table 1).

**Table 1:** Predictors for acute symptomatic seizures

	All epileptic seizures (*n *= 695)	Acute symptomatic (*n* = 170 [24.5%])	Unprovoked (*n* = 525 [75.5%])	Binary logistic regression Exp(B) [95% CI]
Male sex, *n* (%)	452 (65.0)	140 (82.4)	312 (59.4)	3.173 (1.972–5.104)
Age, years, median [IQR]	47.8 [32–62.9]	48.2 [39.5–58.5]	47.7 [29.5–64.9]	n.s
No prior diagnosis of epilepsy, *n* (%)	287 (41.3)	139 (81.8)	148 (28.2)	11.235 (7.198–17.537)
Seizure semiology				
Focal (aware/impaired awareness), *n* (%)	71 (10.2)	6 (3.5)	65 (12.4)	1.000
Bilateral/generalized tonic–clonic, *n* (%)	532 (76.6)	138 (81.2)	394 (75.0)	2.982 (1.172–7.588)
Unclear, *n* (%)	92 (13.2)	26 (15.3)	66 (12.6)	2.535 (0.890–7.221)

*n* number, *IQR* interquartile range, *95% CI* 95% confidence interval, *n.s.* not significant

Taking the results from the logistic regression analysis, we developed a scoring system for estimating the probability of acute symptomatic seizure in the ER (Table 2). The positive predictive value of a seizure with bilateral/generalized tonic–clonic semiology in a male patient without prior diagnosis of epilepsy to be acute symptomatic was 57.1% (95% CI 48.9–64.9).

**Table 2:** Scoring system estimating the probability of acute symptomatic seizure in the ER

Total Score	Positive predictive value for acute symptomatic seizure (95% CI)
0	0.0% (0–10.4)
1	2.6% (0.1–6.3)
2	13.3% (9.0–19.0)
3	33.3% (15.5–56.9)
4	47.3% (36.8–57.9)
5	57.1% (48.9–64.9)

Items to score: male sex: 1 point. Generalized/bilateral tonic–clonic seizure semiology: 1 point. No prior diagnosis of epilepsy: 3 points

*CI* confidence interval

### Causes of ASS and US

**Fig. 2:**
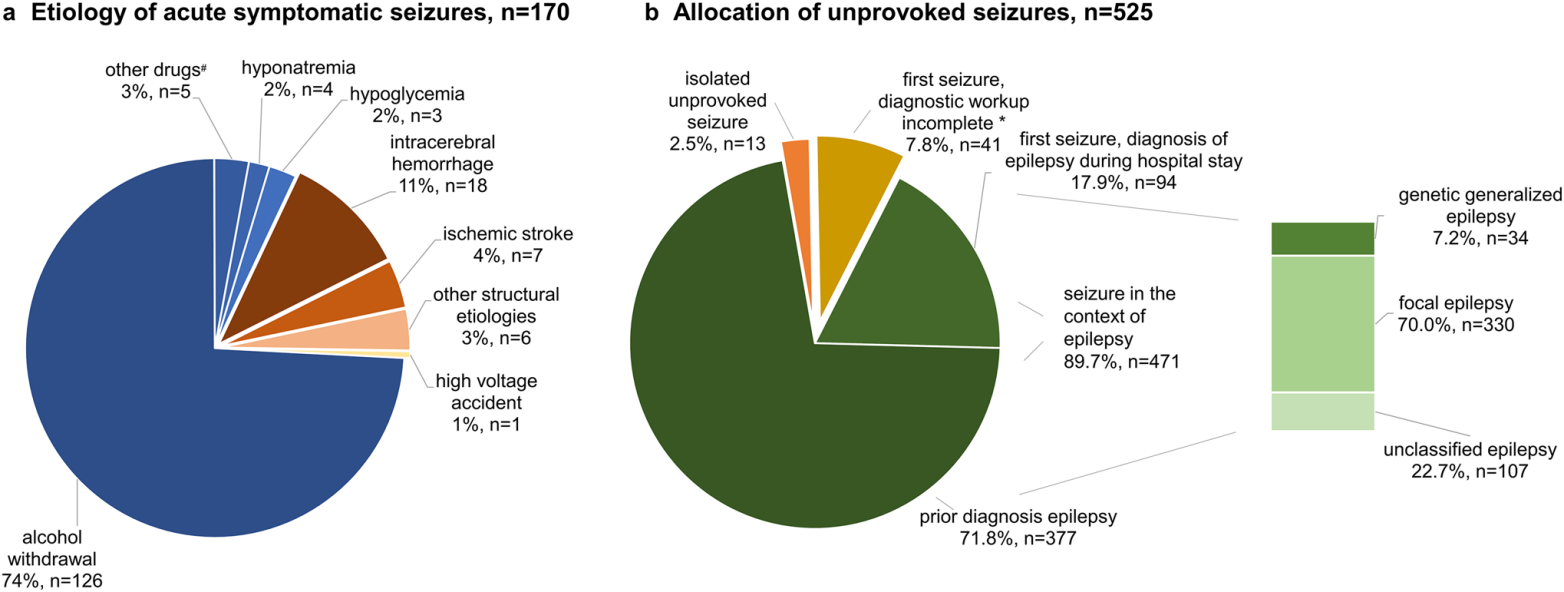
Etiology of acute symptomatic seizures and allocation of unprovoked seizures. **a** Shows the etiologies of ASS. Metabolic–toxic disturbances are shown in blue colors, structural causes are in red tones. **b** Displays the allocation of unprovoked seizures. The bar graph illustrates the classification of established epilepsies. *n *number. ^#^Other drugs include intoxication with gamma-hydroxybutric acid and withdrawal of benzodiazepines. *This includes 34 patients who presented with a first seizure and seven patients, where it remained unclear, whether the seizure was isolated or recurrent. *PPV* positive predictive value, *NPV* negative predictive value, *CI* confidence interval

ASS were caused by metabolic–toxic disturbances in 138 cases (81.2%), alcohol-withdrawal was most common (126 patients, 74.1% of all ASS), other reasons were withdrawal from or intoxication with different drugs, such as benzodiazepines and gamma-hydroxybutric acid. Other rare metabolic causes were hyponatremia and hypoglycemia. The remaining 32 ASS (18.8% of all ASS) were due to cerebral insults (intracerebral hemorrhage and ischemic stroke) and other structural etiologies (brain surgery, meningoencephalitis, posterior reversible encephalopathy syndrome, post-hypoxic encephalopathy, and traumatic brain injury). One seizure was due to a high voltage accident. For visualization of details, see Fig. 2a.

Patients with ASS related to alcohol-withdrawal compared to those with ASS due to all other etiologies were more often male (87% vs. 67%; *p* < 0.003), were younger (47 years vs. 62 years, *p* < 0.001) and more often previously had seizures (69% vs. 23%; *p* < 0.001).

Of the 525 patients with US, 13 patients (2.5% of all US) were considered to have had an isolated US, i.e., they had a first US and MRI and EEG showed no relevant pathologies which would indicate an increased risk of seizure recurrence. In 41 patients with first US (7.8% of all US), the diagnostic workup was incomplete, i.e., the patients were discharged before MRI and/or EEG had been performed, preventing the further allocation of the seizure to an isolated event or to epilepsy. Four hundred and seventy-one patients (89.7%) had US in the context of epilepsy (Fig. 2b), in 377 cases, the diagnosis had been established before presentation to the ER, in 94 cases, the diagnosis was made after the diagnostic workup following the seizure leading to inclusion in this study.

### Diagnostic procedures

**Fig. 3:**
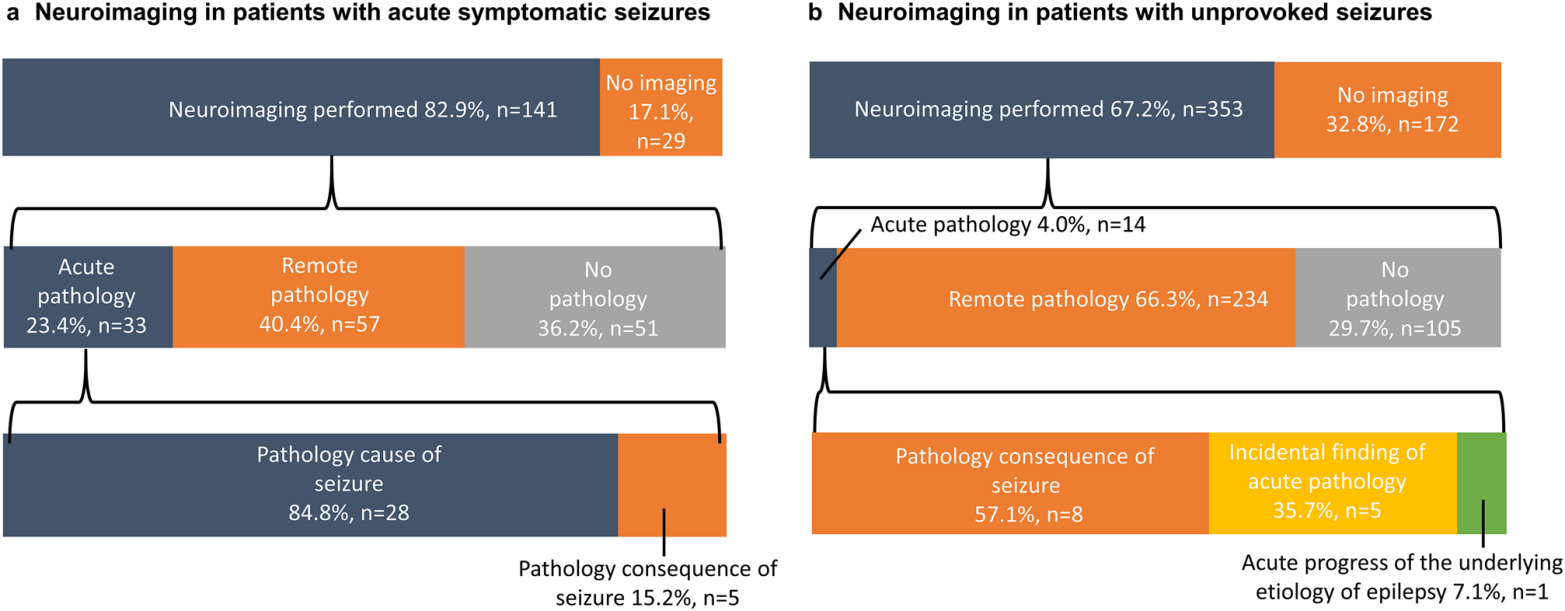
Neuroimaging in patients with acute symptomatic and unprovoked seizures. **a**, **b** Show the proportion of patients that received neuroimaging (first row), the findings of the performed neuroimaging (second row) and the causal relationship of acute pathologies with respect to the seizure (third row). *n* number

Neuroimaging was performed in 494 patients (71.1%) during the hospital stay. Three hundred and forty-three patients received a CT, 54 patients an MRI, and 97 patients received both. In 141 patients with the final diagnosis of ASS and in 353 patients with the final diagnosis of US, neuroimaging was performed (82.9% vs. 67.2%, *p* < 0.001). All patients who eventually had ASS underwent neuroimaging within 24 h of presentation to the ER. An acute pathology was seen in 47 out of 494 patients with neuroimaging (9.5%). In patients with ASS, imaging revealed an acute pathology in 33 cases (23.4% of those with imaging) and a remote pathology in 57 cases (40.4%). In 28 of the 33 patients with ASS, the acute pathology was considered the underlying cause for the ASS, while it was the consequence of the epileptic fit such as intracranial hemorrhage in 5 cases (see Fig. 3a).

In patients with a final diagnosis of US, imaging revealed an acute pathology in 14 cases (4.0% of US with neuroimaging). This was significantly less often than in patients who were eventually considered to have had an ASS (23.4%; *p* < 0.001). In the 8 out of 14 patients with acute pathology, it was considered a consequence of the seizure. In 5 patients, the pathology was considered an incidental finding not related to the seizure (4 patients with subcortical cerebral ischemia, 1 patient with overdrainage of a ventriculo-peritoneal shunt), one patient had an acute progress of the etiology underlying the epilepsy (see Fig. 3b).

### Clinical course and therapeutic intervention

A total of 91 patients (13.1%) received acute ASM such as lorazepam and diazepam in the prehospital phase and 254 patients (36.5%) received ASM in the ER (mostly lorazepam and clobazam). Of these, 47 patients received ASM both in the prehospital phase and in the ER.

In the prehospital phase, acute ASM were administered in 29 patients with a final diagnosis of ASS (17.1% of all patients with ASS) and in 62 patients with a final diagnosis of US (11.8% of US; *p* = 0.078).

In the ER, 79 patients (11.4%) had a seizure recurrence. There was no statistically significant difference in early seizure recurrence between patients with ASS and US (13.5% vs. 10.7%; *p* = 0.307). There was also no statistically significant difference in seizure recurrence in the ER between ASS related to alcohol withdrawal and ASS due to other causes (14.2% vs. 11.6%, *p* = 0.673).

In the ER, acute ASM were given to 95 patients with the final diagnosis of ASS (55.9%) and to 159 patients who eventually were considered to have had an US (30.3%, *p* < 0.001).

**Fig. 4:**
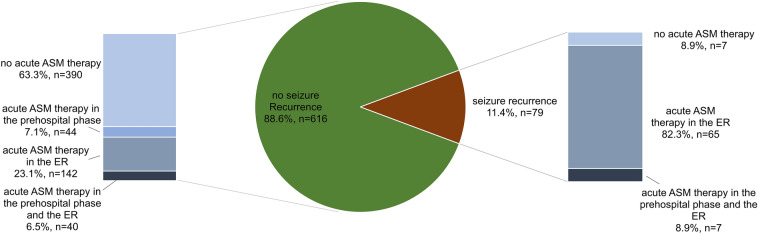
ASM therapy in patients with and without seizure recurrence. The pie chart illustrates frequency of seizure recurrence. The left bar graph shows ASM therapy in patients without seizure recurrence, the right bar graph demonstrates ASM therapy in patients with seizure recurrence. *n* number

Figure 4 shows medical treatment in patients with and without seizure recurrence. Of the 616 patients without seizure recurrence, 226 (33.6%) received ASM therapy in the prehospital phase and/or the ER. Of the 79 patients with seizure recurrence, 72 (91.2%) received ASM therapy in the ER (seven of these patients also had received ASM therapy in the prehospital phase).

## Discussion

In this study, we analyzed ASS as compared to US in the ER with respect to predictors, patient characteristics, diagnostic procedures, and acute treatment. The results of this study are important for everyday clinical management as they can help differentiating ASS from US in the ER, which is essential regarding acute diagnostic workup and decisions on further management of the underlying condition.

One in four patients presenting with an epileptic fit had ASS, all other patients had US. As our main outcome, we identified the variables male sex, no prior diagnosis of epilepsy, and bilateral/generalized tonic–clonic semiology to be independently associated with ASS. The association between male sex and ASS has been described previously [7]. The likely explanation is that most common causes of ASS (alcohol withdrawal, ischemic and hemorrhagic stroke) predominate in male patients [8, 9]. ‘No prior diagnosis of epilepsy’ was the strongest predictor for ASS. Epilepsy is per definition associated with recurrent US. An independent association between prior diagnosis of epilepsy and US, or vice versa no prior diagnosis of epilepsy and ASS is, therefore, plausible. It is noteworthy that the information on prior diagnosis of epilepsy in some cases is not readily available when treating a patient in the ER as the patient maybe unable to give this information during postictal desorientation. This stresses the importance of contacting the patients’ family, next of kin etc. to attain information on possibly pre-existing epilepsy. The association between ASS and a bilateral/generalized tonic–clonic semiology is more difficult to interpret. It may partly be explained by the high proportion of alcohol withdrawal seizures which are mostly considered to be generalized tonic–clonic [10]. Another possible explanation for the dominance of bilateral/generalized tonic–clonic seizures in the ASS group could be that most patients with unprovoked seizures had a focal epilepsy and thus focal aware and impaired awareness seizures.

The variables identified to be associated with ASS have a moderate diagnostic merit: the positive predictive value of a bilateral/generalized tonic–clonic seizure in a male patient without prior diagnosis of epilepsy to be acute symptomatic is 57%. The simple score that we developed can help identifying patients in whom a thorough search for an acute underlying condition is reasonable. A validation of this score in an external cohort would be desirable.

Approximately 80% of ASS were due to metabolic–toxic disturbances, almost three quarters of all ASS were related to alcohol withdrawal. Around 20% of ASS cases were caused by an acute structural lesion. The dominance of seizures related to alcohol withdrawal in this study is striking. This finding is in accordance with a study on 472 patients in Denver, the rate of ASS due to alcohol withdrawal was 59% [11]. A lower rate of only 14% ASS due to drug withdrawal was seen in an analysis of 692 patients in Rochester County in Minnesota covering the years 1935–1984 [7]. The lowest rate was reported in a study from Hong Kong, where alcohol/drug withdrawal was the cause in only 3.8% of ASS in a total of 155 patients [12]. These low rates of alcohol-withdrawal related seizures may be explained by the overall lesser amount of alcohol consumption in both the United States and especially Asia as compared to Germany [13]. Multicenter studies would be desirable to gain further insight in the proportion of different causes for ASS.

Hemorrhagic and ischemic stroke were the most common structural causes of ASS. The risk of ASS is 4–16% in hemorrhagic stroke patients [14, 15] and 3–6% following ischemic stroke [14, 16]. Most ASS occur within 24 h after the cerebrovascular event [14, 17]. Still, it is likely that a significant proportion of ASS after stroke is not seen in the ER as the patients have already been admitted to a stroke unit when the ASS occurs.

Around 80% of patients with ASS and two thirds of patients with US received timely neuroimaging. The question which patient should receive cerebral imaging and which patient should not is difficult to answer. Roughly 1 in 10 patients who received neuroimaging had an acute pathology, but the diagnostic yield of neuroimaging varied drastically between ASS (20%) and US (4%). In only 2% of patients with US who underwent neuroimaging, an acute pathology was detected that was considered a consequence of the seizure. Even though most injuries caused by epileptic seizures are minor [18], there is evidence that neurological examination is not sufficiently predictive of traumatic brain injuries [19]. This even led to the suggestion that after an epileptic seizure every patient should get a CT scan in the ER [19]. An analysis of data on 381 patients who received neuroimaging after a recurrent (non-index) unprovoked seizure showed pathological findings in 59% of cases. In 3% of cases, neuroimaging led to a treatment change. An increased diagnostic yield of neuroimaging was associated with the clinical parameters acute head trauma, prolonged impaired consciousness, and focal neurological deficits [20]. Taken together, the findings of the aforementioned study as well as the variables that were independently associated with ASS in our study should be considered when deciding whether to request acute neuroimaging or not after an epileptic seizure.

Whether to treat a patient with acute ASM or not is the final relevant question when approaching a patient after an epileptic fit in the ER. In addition, there is no indication for permanent treatment with ASM after an ASS as seizure recurrence rates are overall low [3]. Following a first US, permanent ASM therapy is usually justified when either EEG or MRI examination show signs of an increased risk of further epileptic fits, thus defining epilepsy [21]. In the ER, acute ASM were administered in 56% of the current patients with ASS and in 30% of the patients with US. With more than 10%, the seizure recurrence rate was high in patients with ASS and US. In a study on almost 500 children with a first seizure (about one quarter of which had an ASS), 14% had a recurrence within 24 h [22]. It is important to consider that in our study population approximately 90% of US occurred in the context of epilepsy, which is per definition associated with an inherent risk of seizure recurrence.

More than 90% of the current patients received ASM therapy after seizure recurrence in the ER. Interestingly, also 30% of patients without seizure recurrence were treated with an ASM in the ER. Due to the retrospective nature of the study, no scientifically sound assertions can be made with respect to the effectiveness of treatment with ASM. In a randomized controlled trial, the efficacy of acute antiseizure treatment after ASS due to alcohol withdrawal has been demonstrated; the administration of 2 mg i.v. lorazepam was associated with a significant reduction of seizure recurrence in the first 6 h after the index seizure (3% vs. 24% recurrence rate in the placebo group) [23].

There are limitations to consider. Although our study includes two ERs covering heterogeneous parts of the city, it would be desirable to analyze data from more centers which would increase generalizability of our findings. In addition, the dominance of seizures related to alcohol withdrawal could potentially limit the informative value on ASS of other etiologies. Finally, even though we excluded 102 patients with confirmed non-epileptic seizures and in addition 132 patients, where evidence extracted from the medical charts was not sufficient for post-hoc confirmation of an epileptic seizure, we cannot entirely exclude that single patients in our cohort eventually have had a psychogenic non-epileptic seizure or syncope. In a previous study, a fraction of almost 14% of seizures in the ER was misdiagnosed [24]. However, in the current cohort, diagnosis of epileptic seizures was reassessed by the authors, not only based on information gathered in the ER but, if available, also on findings collected during the subsequent hospital stay.

In summary, we characterized ASS and US in two large ERs. ASS were independently associated with male sex, no prior diagnosis of epilepsy, and bilateral/generalized semiology. Neuroimaging was performed more often in patients with a final diagnosis of ASS than in those after US. The same was true for administration of acute ASM. The results of this study can help identifying patients with ASS in the ER which is important as ASS bear relevant consequences regarding further diagnostic procedures and treatment. It would be desirable to expand our study to a prospective design in more ERs at different, heterogeneous locations.

## Data Availability

The data that support the findings of this study are available on request from the corresponding author. The data are not publicly available due to privacy or ethical restrictions.
